# Case report: PLPHP deficiency, a rare but important cause of B6-responsive disorders: A report of three novel individuals and review of 51 cases

**DOI:** 10.3389/fneur.2022.913652

**Published:** 2022-10-17

**Authors:** Sarah Alsubhi, Bradley Osterman, Nicolas Chrestian, François Dubeau, Daniela Buhas, Myriam Srour

**Affiliations:** ^1^Division of Pediatric Neurology, Department of Pediatrics, McGill University, Montreal, QC, Canada; ^2^Department of Pediatric Neurology, Pediatric Neuromuscular Disorder, Centre Mère Enfant Soleil, Laval University, Quebec City, QC, Canada; ^3^Department of Neurology and Neurosurgery McGill University, Montreal, QC, Canada; ^4^Division of Medical Genetics, Department of Specialized Medicine, McGill University Health Center, Montreal, QC, Canada; ^5^Department of Human Genetics, McGill University, Montreal, QC, Canada; ^6^Child Health and Human Development Program (CHHD), McGill University Health Center Research Institute, Montreal, QC, Canada

**Keywords:** PLPBP, PROSC, pyridoxine, B6, PLPHP, seizures, status epilepticus, B6RDs

## Abstract

PLPHP (pyridoxal-phosphate homeostasis protein) deficiency is caused by biallelic pathogenic variants in *PLPBP* and is a rare cause of pyridoxine-responsive disorders. We describe three French-Canadian individuals with PLPHP deficiency, including one with unusual paroxysmal episodes lacking EEG correlation with a suspicious movement disorder, rarely reported in B6RDs. In addition, we review the clinical features and treatment responses of all 51 previously published individuals with PLPHP deficiency. Our case series underlines the importance of considering *PLPBP* mutations in individuals with partially B6-responsive seizures and highlights the presence of a founder effect in the French-Canadian population.

## Introduction

Vitamin B6-responsive disorders (B6RDs) are a heterogeneous group of autosomal recessive conditions characterized by neonatal-onset seizures that are resistant to anti-seizure medications (ASMs) but uniquely responsive to pyridoxine (vitamin B6) or its active form, pyridoxal-5'-phosphate (PLP). PLP is essential for normal brain function, given its role as a cofactor in more than 160 enzymatic reactions, including those involved in glucose, lipid, and amino acid metabolism and neurotransmitter synthesis (1).

The most common B6RD is pyridoxine-dependent epilepsy-ALDH7A1, caused by biallelic pathogenic variants in *ALDH7A1*, which encodes for the alpha-aminoadipic semialdehyde dehydrogenase, antiquitin (2). PLP deficiency may result from dysfunction of several other genes, either through PLP inactivation or disruption of vitamin B6 metabolism (1, 3–5) (Supplementary Figures 1A,B and Supplementary Table 1 for PLP synthesis pathways and summary of main B6RDs). *PLPBP* (previously *PROSC*) encodes for Pyridoxal-Phosphate Homeostasis Protein (PLPHP), which is involved in the homeostatic regulation of free PLP levels (6, 7).

We describe three French-Canadian individuals with PLPHP deficiency and review the clinical features and treatment responses of all 51 previously published cases (1, 6–16). Our report underlines the importance of considering *PLPBP* mutations in individuals with partially B6-responsive seizures and highlights the presence of a founder effect in the French-Canadian population. In addition, we describe the unusual presentation of paroxysmal events lacking EEG correlation with a suspicious movement disorder, rarely reported in B6RDs.

## Case descriptions

### Subject 1

This 37-year-old man was born to non-consanguineous French-Canadian parents from the Saguenay-Lac-Saint-Jean region of Quebec. Family and perinatal histories are unremarkable. He presented at the age of 2 weeks with frequent clusters of brief flexion spasms lasting between 15 minutes to 2 hours, associated with apnea and worsening lethargy. An EEG revealed background slowing and active multifocal epileptic abnormalities. The events were considered epileptic and were unresponsive to nitrazepam, phenytoin, and prednisolone. Administration of intravenous (IV) pyridoxine resulted in the cessation of the spasms and EEG normalization.

The patient was diagnosed with presumed pyridoxine-dependent epilepsy (PDE) and was prescribed pyridoxine supplementation combined with multiple ASMs throughout his life. He continued to have paroxysmal events 1–3 times per month that were considered epileptic. These were relatively stereotyped, lasting 1 to 2 min, and consisted of facial grimacing, tonic posturing of the upper arms, eye blinking with or without staring, hand automatisms, and hyperkinetic movement. He experienced several episodes of exacerbation during which the events occurred in clusters, lasting 15–18 hours and requiring hospitalization. These exacerbations were associated with nausea and vomiting and were usually triggered by fatigue and stress. EEGs performed during these events were always normal.

The patient gained early developmental milestones appropriately, though he was clumsy and had mild gait unsteadiness. He has average intelligence and earned a college diploma. He now works as a library technician. Neurological examinations consistently documented nystagmus, dysarthria, truncal titubation, dysmetria, tremors, and difficulty performing tandem gait.

In terms of investigations, markers for *ALDH7A1*-related PDE, serum pipecolic acid, and urine for α-aminoadipic semialdehyde (α-AASA) were measured repeatedly and were consistently normal. Molecular sequencing of *ALDH7A1* was normal. Metabolic investigations were unremarkable aside from persistent high plasma glycine and low arginine levels. Chromosomal microarray and multiple brain MRIs were normal.

At 35 years, the patient took 1,200 mg/day of pyridoxine combined with valproic acid, carbamazepine, clonazepam, and levetiracetam. Because of the incomplete response to pyridoxine, normal EEGs, uncertainty in diagnosis, and concern of acquiring a neuropathy associated with high dose pyridoxine treatment (2), pyridoxine was gradually weaned off over 2 years. One month after complete pyridoxine cessation, the patient's clinical status rapidly deteriorated. He developed episodes of paroxysmal dizziness, vomiting, and nausea, occurring eight times daily. He lost 40 lb over 3 weeks. His regular paroxysmal episodes increased to over 50 per day, prompting hospitalization and treatment as probable status epilepticus. There was no response to IV ASMs. Video EEG captured multiple clinical events with no electrographic correlation (Supplementary Video 1). No interictal epileptiform abnormality was observed. Administration of IV pyridoxine resulted in a dramatic decrease in the number of events on the 1st day and resolution of the motor and gastrointestinal manifestations on subsequent days. Our patient regained his usual clinical status on pyridoxine 200 mg twice a day.

An epilepsy gene panel (GeneDx) comprising over 1,500 genes, including those involved in B6 metabolism, was performed on the patient and his unaffected parents. Our patient carries two rare compound heterozygous variants in *PLPBP* (MIM^*^ 604436, NM_007198.3, c.370_373delGACA [p. Asp124LysfsX2] and c.704 T>G [p.Val235Gly]). The c.704 T>G [p.Val235Gly] missense variant is very rare (mean allele frequency (MAF) = 0.0001309 in gnomAD database), predicted disease-causing by multiple in silico tools [Mutationtaster, SIFT, and Provean (17–19) and reported in ClinVar in one patient with early onset PDE (VCV000802398.1)]. The truncating frameshift variant, c.370_373delGACA [p.Asp124LysfsX2] is also rare (MAF = 0.00007185 in gnomAD) and has been previously reported as disease-causing in a homozygous state in eight individuals, five of whom are of French-Canadian origin (1, 8, 15). We interpreted these two variants as pathogenic. Given the reported improvement with folinic acid supplementation in some patients with PLPHP deficiency, folinic acid was prescribed to our patient without clear clinical benefit.

### Subject 2

This 19-year-old man was born to non-consanguienous parents from the Saguenay-Lac-Saint-Jean region of Quebec by repeat C-section following an uneventful pregnancy. Birthweight was 4.2 kg (+1 SD); length, 50 cm (0 SD); and head circumference, 37.5 cm (+2 SD).

He developed seizures, excessive irritability, desaturations, and metabolic lactic acidosis on the 1st day of his life. The interictal EEG on day 1 was normal. Seizures were refractory to ASMs but ceased after administration of a single dose of pyridoxine 50 mg IV on day 13 of life. Metabolic investigations revealed high plasma and CSF glycine, with a normal CSF glycine/plasma ratio. Brain MRI revealed delayed myelination, diffuse gyral simplification, enlargement of the pericerebral spaces, periventricular cystic lesions in the left anterior frontal horn, and abnormal high T2 and low T1 signal abnormalities in bilateral putamina. He was diagnosed with probable B6RDs and discharged on pyridoxine and ASMs.

The patient's seizures were never fully controlled, and his ASMs were frequently adjusted. Seizures consist of generalized stiffness and multifocal clonic movements lasting 3–4 min and occurring every 2–8 weeks. Weaning of pyridoxine was attempted at age 2.5 years, leading to a seizure exacerbation requiring hospitalization and re-introduction of pyridoxine. The patient was hospitalized twice (ages 4 and 6 years) for status epilepticus with noticeable pre-ictal vomiting and constipation.

Early in life, the patient had significant gastroesophageal reflux treated with medication. His development is globally delayed. He sat with support at 2 years, stood with support at 2.5 years, but never walked. He is wheelchair-bound, non-verbal, and has a severe intellectual disability.

Physical examinations documented microcephaly (-2.1SD), axial hypotonia, and appendicular paratonia. Non-purposeful hand movements and dystonic posturing for the upper limbs were noted on multiple visits.

**Figure 1:**
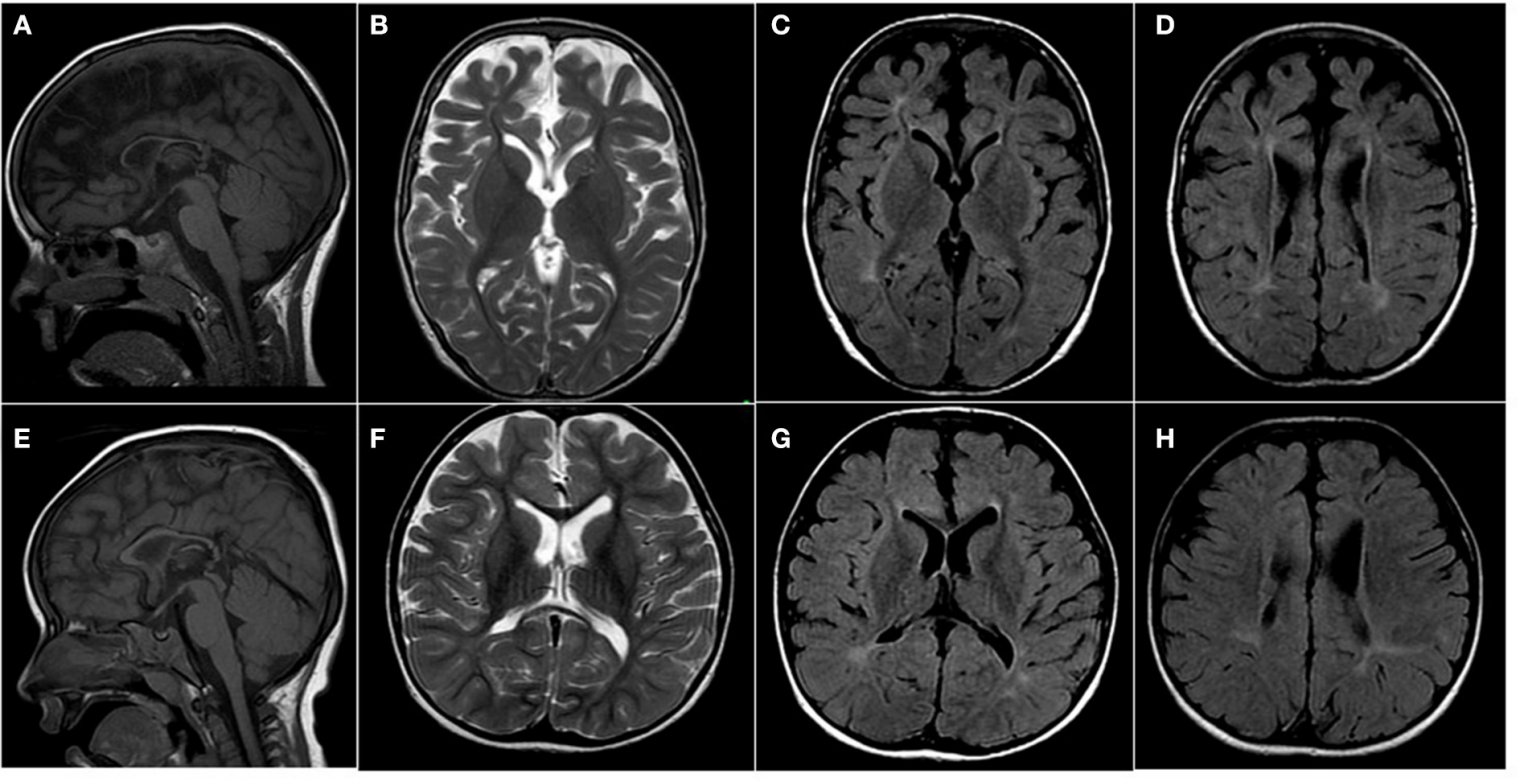
Brain imaging of subjects with PLPHP deficiency. MRI of subjects 2 **(A–D)** and 3 **(E,F)** at 4.5 and 2.5 years. **(A,E)** Sagittal T1 demonstrates thin corpus callosum. **(B,F)** Axial views show a patchy bilateral increase in T2 and **(C,D,G,H)** FLAIR signal in the deep white matter around the ventricles at bilateral frontal and bilateral peritrigonal regions. Both subjects have cerebral atrophy, most noted in subject 2, with a prominence of CSF spaces, particularly over frontal and sylvian regions bilaterally.

MRI brain imaging (at 3.5 and 4.5 years) showed progressive cerebral atrophy with enlargement of ventricles and extra-axial CSF, corpus callosum thinning, and non-specific bilateral patchy high T2/FLAIR signal abnormalities in the periventricular and deep white matter (Figures 1A–D). The periventricular cystic lesions noted in the neonatal period were no longer visible on follow-up imaging.

Genetic tests were normal, including karyotype, chromosomal microarray, sequencing of *ALDH7A1, PNPO*, and mitochondrial DNA sequencing. Targeted testing of the frameshift pathogenic variant c.370-373del (p.Asp124Lysfs^*^2) in *PLPBP* identified in his similarly affected brother, subject 3, confirmed its presence in a homozygous state. PLP (100 mg three times a day) was added to his regimen of pyridoxine (100 mg twice daily), levetiracetam, and clonazepam. This did not result in any clear change in the frequency of seizures, but these are now briefer.

Although parents have noticed no significant change in cognitive function since the start of PLP, seizures have become briefer and slightly less frequent, from once every 2–8 weeks to once every 2 months. At age 17, EEG showed active multifocal epileptic abnormality with left posterior temporoparietal predominance.

### Subject 3

He is the younger brother of subject two and is currently 16 years old. The antenatal course was uneventful. The patient was born at 40 weeks of gestation by repeated C-sections. APGAR scores were average. His birthweight was 3.68 kg (+0.5SD), and his head circumference was 36.5 cm (+1.5SD).

He experienced seizures in the 1^st^ hour of life, described as tonic rigidity with gaze fixation, clonic jerking of the limbs, and apnea. He also had metabolic lactic acidosis. CSF lactate was elevated. Seizures ceased after treatment with diazepam, phenobarbital, and pyridoxine. Initial EEG showed burst suppression. However, the background improved the following week, showing multifocal epileptic abnormalities. Brain MRI at the age of 2 days showed diffuse hypomyelination. Seizures recurred on the day of life 10, and the B6 dose was readjusted. He was discharged at age 2 weeks on pyridoxine and clonazepam.

The patient's seizures have only been partially controlled, occurring every 1–2 weeks with increased frequency during illness and sleep deprivation. They are characterized by gaze fixation, and impaired awareness, with or without progression to bilateral tonic-clonic movements. During the 1st year of life, the EEG background was normal. However, more recent testing has revealed a diffuse disturbance of cerebral activity in the form of a poor anterior-posterior gradient and posterior dominant rhythm for age. An active focal epileptic abnormality was noted in the left parietal region. Pyridoxine was always combined with ASMs. The dose was gradually increased from 50 mg twice daily to 100 mg twice daily.

The patient is non-verbal and has a severe intellectual disability. He walked at the age of 2 years and remains ambulatory. He is followed by psychiatry for hyperactivity and behavioral issues. He was also treated for gastroesophageal reflux until the age of 8 years.

A brain MRI at 2.5 years showed decreased myelination and non-specific hyper-T2/FLAIR signal abnormalities in the periventricular white matter regions, posteriorly extending to the U-fibers. The CSF spaces and ventricles were enlarged, suggesting poor cerebral growth (Figures 1E–H).

Karyotype, chromosomal microarray, and mitochondrial DNA gene sequencing were normal. Single gene sequencing for the *PLPBP* gene revealed a homozygous pathogenic variant (NM_007198.3:c.370-373del, p.Asp124Lysfs^*^2). This variant was also identified in subject 1.

Following his molecular diagnosis, he was also prescribed PLP 100 mg three times a day. No significant change in cognition or seizure frequency has been noted following the introduction of PLP, although seizure duration has slightly decreased.

### Literature review

We performed a literature review of all previously published cases of PLPHP deficiency by searching for the terms “PROSC,” “PLPBP,” and “PLPHP” in PubMed. In total, 12 articles were identified describing 51 individuals with PLPHP deficiency (1, 6–16). We also obtained updated clinical information of the patients previously reported by Maitou et al. (8). We reviewed the clinical and biochemical features and treatment responses of all cases, including our three patients (a total of 54 individuals with PLPHP deficiency). These are summarized in Table 1.

**Table 1:** Summary of clinical features in individuals with *PLPBP* pathogenic variants.

	**This report**	**Darin et al. (6)**	**Plecko et al. (16)**	**Kernohan et al. (15)**	**Initiative et al. (14)**	**Shiraku et al. (13)**	**Johnstone et al. (1)**	**Jensen et al. (7)**	**Johansen et al. (12)**	**Koul et al. (11)**	**Heath et al. (10)**	**Ahmed et al. (9)**	**Maitou et al. (8)**	**Total (%)**
Number of patients	3	7	4	1	1	4	12	2	1	12	1	1	5	54
Deceased (age at death)	0/3	1/7 (4 m)	0/4	1/1 (2 m)	0/1	0/4	2/12 (2 w, 8 w)	1/2 (7 w)	0/1	0/12	0/1	0/1	3/5 (2 w, 1 m, 16 m)	8/54 (15)
Suspected fetal seizures	1/3	3/7	0/4	0/1	0/1	1/ 4	2/12	0/2	0/2	2/12	0/1	0/1	0/5	9/54 (17)
**Symptoms at initial presentation**														
Seizures	3/3	7/7	4/4	1/1	NR	4/4	11/12	2/2	1/1	12/12	1/1	1/1	5/5	52/54 (96)
Neonatal onset (<28 d)	3/3	6/7	4/4	1/1	NR	2/4	11/12	2/2	1/1	12/12	1/1	1/1	5/5	49/54 (91)
Infantile onset (28 d- to 2 y)	0/3	1/7	0/4	0/1	NR	2/4	0/12	0/2	0/1	0/12	0/1	0/1	0/5	3/54 (6)
Movement disorder	0/3	0/7	0/4	0/1	NR	0/4	1/12	0/2	0/1	0/12	0/1	0/1	0/5	1/54 (2)
GI symptoms	0/3	3/7	0/4	NR	NR	0/4	0/12	2/2	0/1	0/12	0/1	0/1	0/5	5/54 (9)
Burst suppression on EEG	1/3	5/7	1/ 4	NR	NR	1/ 4	5/11	0/2	1/1	2/12	1/1	0/1	4/5	21/54 (39)
**Initial response to B6/PLP treatment**														
B6 and/or PLP trial attempted	3/3	7/7	4/4	NR	NR	4/4	10/12	1/2	1/1	12/12	1/1	1/1	5/5	49/54 (91)
B6 trial	3/3	7/7	4/4	-	-	3/4	9^a^/10	1/1	1/1	12/12	1/1	1/1	2/5	44/49 (90)
Complete resolution of seizures	3/3	7/7	3/4	-	-	1/3	7/9	1/1	1/1	12/12	0/1	1/1	0/2	36/44 (82)
Partial improvement	0/3	0/7	0/4	-	-	1/3	0/9	0/1	0/1	0/12	1/1	0/1	2/2	4/44 (9)
No effect	0/3	0/7	1/4	-	-	1/3	2/9	0/1	0/1	0/12	0/1	0/1	0/2	4/44 (9)
PLP trial	0/3	0/7	0/4	-	-	1/4	2^a^/10	0/2	0/1	0/12	0/1	0/1	3/5	6/49 (12)
Complete resolution of seizures	-	-	-	-	-	1/1	2/2	-	-	-	-	-	0/3	3/6 (50)
Partial improvement	-	-	-	-	-	0/1	0/2	-	-	-	-	-	0/3	0/6 (0)
No effect	-	-	-	-	-	0/1	0/2	-	-	-	-	-	3/3	3/6 (50)
**Maintenance therapy and response**														
Seizure-free	0/3	2/6	3/4	NR	NR	3/4	8/10	0/1	1/1	11/12	1/1	1/1	1/2	31/45 (69)
B6 only (# sz-free)	0/3	1/6 (1^b^)	3/4 (2)	NR	NR	1/4 (1)	5/10 (3^b^)	0/1	0/1	1/12 (1)^c^	0/1	0/1	0/2	11/45 (24); sz-free: 8/45 (18)
B6 +/−ASM (# sz-free)	1/3	1/6 (1^b^)	1/4 (1)	NR	NR	2/4 (1^b^)	2/10 (2^b^)	0/1	1/1 (1)	11/12^d^ (10)	0/1	0/1	0/2	19/45 (42) sz-free: 16/45 (36)
PLP + ASM (# sz-free)	0/3	4/6	0/4	NR	NR	1/4 (1)	1/10 (1)	1/1	0/1	0/12	1/1 (1)	1/1 (1^b^)	1/2	10/45 (22); sz-free: 4/45 (9)
PLP + B6 alone (# sz-free)	0/3	0/6	0/4	NR	NR	0/4	1/10 (1)	0/1	0/1	0/12	0/1	0/1	1/2 (1)	2/45 (4); sz-free: 2/45 (4)
PLP + B6 +ASM (# sz-free)	2/3	0/6	0/4	NR	NR	0/4	0/10	0/1	0/1	0/12	0/1	0/1	0/2	2/45 (4); sz-free: 0/45 (0)
PLP + Folinic acid (# sz-free)	0/3	0/6	0/4	NR	NR	0/4	1/10 (1^b^)	0/1	0/1	0/12	0/1	0/1	0/2	1/45 (2): sz-free: 1/45 (2)
**Switch from B6 to PLP (+/−ASM)** Complete sz resolution Partial improvement No effect	0/3 - - -	4/6 0/4 4/4 0/4	0/4 - - -	NR - - -	NR - - -	0/2 - - -	2/7 1/2 0/2 1/2	1/1 0/1 1/1 0/1	0/1 - - -	0/12 - - -	1/1 1/1 0/1 0/1	1/1 1/1 0/1 0/1	0/1 - - -	9/39 (24) 3/9 (33) 5/9 (56) 1/9 (11)
**GDD, ID or ASD**	2/3	6/6	3/4	NR	NR	4/4	7/10	1/ 1	1/1	2/12	0/1	1/1	3/3	30/46 (65)
**Movement disorder**	2/3	0/7	0/4	0/1	0/1	1/4	1/12	0/2	0/1	0/12	0/1	0/1	0/5	4/54 (7)
**Microcephaly**	2/3	6/7	0/4	NR	NR	3/4	2/12	2/2	0/1	NR	1/1	1/1	3/5	20/54 (37)
**Brain MRI**														
Abnormal Normal Not available	2/3 1/3 -	4/7 3/7 -	0/4 4/4 -	NR NR 1/1	NR NR 1/1	3/4 1/4 -	7/12 4/12 1/12	2/2 0/2 -	0/1 1/1 -	1/12 2/12 9/12	1/1 0/1 -	1/1 0/1 -	5/5 0/5 -	26/42 (62) 16/42 (38) 12/54 (22)
**Biomarkers**														
Lactic acidosis (CSF or plasma)	2/3	4/7	0/4	NR	NR	0/ 4	6/12	2/2	0/1	NR	1/1	1/1	5/5	21/54 (39)
↑plasma glycine, threonine, or serine Other CSF abnormalities^e^	3/3 2/3	1/7 2/7	1/ 4 1/ 4	NR NR	NR NR	1/ 4 1/ 4	3/12 3/12	2/2 1/ 2	0/1 0/1	NR NR	1/1 1/1	1/1 1/1	0/5 0/5	13/54 (24) 12/54 (22)

GI, Gastrointestinal; ID, Intellectual disability; PLP, Pyridoxal-5'- phosphate; m, month; PLP, Pyridoxal-5'- phosphate; sz, Seizure; w, week; ^a^, One patient received a trial of both B6 and PLP; ^b^, Seizure free except breakthrough seizures during febrile illness; ^c^, the patient became seizure-free after addition of folinic acid; ^d^, Unclear whether patients were on ASMs in addition to pyridoxine; ^e^, CSF abnormalities aside from high lactate is ↑ glycine, ↑L-DOPA, ↑3-methyltyrosine, or ↑threonine and Low HVA (Homovanillic acid).

#### Initial presenting features and response to B6 supplementation

Seizures were the initial symptom in almost all individuals (96%, 52/54). Seizures presented in the neonatal period (<28 days) in 91% (49/52). The oldest age at the presentation was 3 months. Abnormal intrauterine movements were reported in 17% (9/54). Initial EEGs were variable, but burst suppression was the most frequent finding (39%, 21/54). At presentation, patients had variable and multiple seizure types, which included myoclonic (23%, 13/52), bilateral tonic-clonic, tonic (15%, 9/52), epileptic spasms (15%, 8/52), clonic (8%, 5/52), and subtle seizures (10%, 5/52). Autonomic features (such as hypertension, tachycardia, vomiting, and abdominal distention) were frequently associated with seizures. A pyridoxine or PLP trial was documented in 91% (49/54) and resulted in initial seizure cessation or improvement in all patients.

Elevated serum and/or CSF lactate, with or without acidosis, was frequently documented at presentation in individuals with PLPHP deficiency (39%, 21/54), with peak concentrations ranging between 4.2 and 21 mmol/l (normal <2.5 mmol/l). In most patients, lactate levels normalized in subsequent days. However, a subset (13%, 7/54) have a neonatal mitochondrial encephalopathy-like presentation, characterized by persistent metabolic lactic acidosis and epileptic encephalopathy. Suspicion of an underlying mitochondrial disorder often delays B6 trial and diagnosis of the B6RDs, leading to delayed or lack of appropriate treatment. Of the seven reported individuals with a neonatal mitochondrial encephalopathy-like presentation, five died early in life (2 weeks-16 months) (1, 8): two died in the neonatal period without receiving a B6 trial, and three died following B6 treatment withdrawal. High glycine in CSF and/or plasma was reported in 24% (13/54) of individuals. Two died before a pyridoxine/PLP trial was considered a mitochondrial disorder, or non-ketotic hyperglycinemia was suspected. Thus, lactic acidosis and hyperglycinemia may represent important diagnostic pitfalls in PLPHP deficiency.

#### Clinical evolution

Despite a dramatic early response to B6/PLP, almost all patients (74%, 34/46) had incomplete seizure control, even with the concomitant use of ASMs. Pyridoxine or PLP withdrawal was attempted in 33% (17/49) and worsened clinical status. In 9 patients, pyridoxine treatment was switched to PLP and resulted in seizure resolution in 3, improved seizure control in 5, and no response in 1, suggesting that a trial with PLP should be attempted in patients with poor or incomplete response to pyridoxine. Interestingly, adding folinic acid to two patients who failed the pyridoxine and PLP trial resulted in prompt seizure cessation (1, 11). The majority of patients who responded to pyridoxine and/or PLP required combined treatment with ASMs (73%, 33/45).

Developmental delay was present in 65% (26/46) of patients, intellectual disability in 27% (7/26), and autism in 23% (6/26). Microcephaly was noted in 37% (20/54, congenital in 4, acquired in 16), cerebellar signs (such as incoordination, dysarthria, and balance instability) in 32% (6/19), and hypotonia in 32% (6/19).

Gastrointestinal dysfunction has been reported in 15% (8/54) of patients with PLPHP deficiency, with symptoms that include abdominal distension, vomiting, feeding intolerance, constipation, hematemesis, and gastroesophageal reflux.

#### Imaging findings

Brain MRI was abnormal in 62% (26/42) and was characterized by the variable presence of white matter signal changes (55%, 23/42), underdeveloped gyri and shallow sulci (43%, 18/42), periventricular or temporal cysts (31%, 13/42) and thin corpus callosum (12%, 5/42).

## Discussion

PLPHP deficiency is a rare but significant cause of B6 responsive seizures. In this report, we describe three patients with biallelic pathogenic variants in *PLPBP* presenting with neonatal-onset seizures that were partially responsive to B6 supplementation, including one individual who had paroxysmal episodes with no clear EEG correlate. In addition, we reviewed the clinical and biochemical features and the response to treatment of 51 additional individuals with molecularly confirmed PLPHP deficiency.

Our three patients presented in the neonatal period with B6-responsive seizures. Following an initial cessation after B6 supplementation, our patients continued to experience seizures despite treatment with B6 and additional ASMs. Incomplete response to B6 was observed in approximately 3/4 of the reported cases. The addition of PLP or folinic acid to B6 therapy resulted in seizure cessation or improved seizure control in a subset of patients, thereby suggesting that all individuals with seizures that are partially responsive to B6 would benefit from a trial of PLP and folinic acid supplementation.

The possibility of a B6-responsive movement disorder is strongly considered in our subject 1, though epileptic seizures lacking EEG correlation cannot be fully excluded. The absence of any change in the EEG background, despite over 50 events per day on telemetry during the patient's admission, is extremely unusual and inconsistent with epilepsy. Movement disorders have rarely been described in association with PLPHP deficiency. Johnstone et al. (1) reported a clinical course similar to subject 1: a 2-month-old child with severe movement disorder consisting of opisthotonos and oculogyric crises, which were resolved with treatment with PLP and pyridoxine. The infant never developed any seizures. Shiraku et al. (13) described a 9-month-old child with a presumed status epilepticus in the context of gastroenteritis who developed dystonia, orobuccal dyskinesias, and eye flickering.

Interestingly, subject 1 had significant gastrointestinal symptoms with B6 withdrawal, and subjects 1 and 2 had nausea and vomiting associated with their ictal events. Autonomic and gastrointestinal symptoms have been frequently described in patients with PLPHP deficiency (Table 1 and literature review). Their pathophysiology is unclear but is possibly related to a disturbance in monoamines neurotransmitters synthesis secondary to the disrupted PLP-dependent activity of Aromatic L-amino acid decarboxylase (AADC), a key enzyme in dopamine and serotonin synthesis (6, 20, 21) (Supplementary Figure 1C).

The previously reported pathogenic variants in *PLPBP* include protein-truncating (non-sense, *n* = 2; frameshift, *n* = 3 and splice site, *n* = 2) and missense (*n* = 16) variants, which have a presumed loss of function mechanism of action (Supplementary Table 2). A clear genotype-phenotype correlation is not readily apparent. The three individuals we describe in this report are of French-Canadian ancestry, from the Saguenay-Lac-Saint-Jean region of Quebec, and carry the same frameshift variant [c.370_373delGACA [p.Asp124LysfsX2] in a homozygous or compound heterozygous state. This variant has been previously described in a homozygous state in five other French-Canadians, and one individual of Cree ancestry. It represents a founder mutation based on haplotype analysis (8). The eight individuals homozygous for this variant are at the severe end of the clinical spectrum with poor outcomes: six presented with a neonatal mitochondrial encephalopathy-like phenotype, and five died early in life (2 weeks to 16 months). Both subjects 2 and 3 have post-natal microcephaly, severe intellectual disability, and are non-verbal. In contrast, subject one was compound heterozygous for the truncating recurrent variant and a missense [c.704 T>G [p.Val235Gly] variant had a favorable cognitive outcome with average intelligence and brain imaging, suggesting that the missense variant is hypomorphic.

In summary, this report and literature review underline the importance of considering PLPHP deficiency in individuals with seizures partially responsive to B6, especially in neonates with drug-resistant seizures and mitochondrial encephalopathy-like presentation. Prompt treatment with a B6-vitamin results in dramatic early seizure improvement, though incomplete seizure control is seen in most patients. The addition of PLP or folinic acid to B6 therapy may be of benefit. Movement disorders, though rare, can be a manifestation of PLPHP deficiency. Finally, there is an important founder effect in the French-Canadian population, with a recurrent truncating pathogenic variant in *PLPBP* associated with a severe clinical phenotype and poor outcomes.

## Data availability statement

The raw data supporting the conclusions of this article will be made available by the authors, without undue reservation.

## Ethics statement

The studies involving human participants were reviewed and approved by McGill University Health Center Research Ethics Board. Written informed consent from the participants' legal guardian/next of kin was not required to participate in this study in accordance with the national legislation and the institutional requirements. Written informed consent was obtained from the individual for the publication of any potentially identifiable images or data included in this article.

## Author contributions

SA: drafting and revising the manuscript for content, including medical writing for content, major role in the acquisition of data, additional contributions, collected clinical data, and drafted the manuscript for intellectual content. BO and NC: drafting and revising the manuscript for content, including medical writing for content, additional contributions, and critically reviewed the manuscript. FD: drafting and revising the manuscript for content, including medical writing for content, major role in the acquisition of data, additional contributions, and critically reviewed the manuscript. DB: drafting and revising the manuscript for content, including medical writing for content and additional contributions, and critically reviewed the manuscript. MS: drafting and revising the manuscript for content, including medical writing for content, major role in the acquisition of data, additional contributions, conceptualization of the study, and critically reviewing the manuscript. All authors contributed to the article and approved the submitted version.

## Conflict of interest

The authors declare that reposting the case was conducted in the absence of any commercial or financial relationships that could be construed as a potential conflict of interest.

## Publisher's note

All claims expressed in this article are solely those of the authors and do not necessarily represent those of their affiliated organizations, or those of the publisher, the editors and the reviewers. Any product that may be evaluated in this article, or claim that may be made by its manufacturer, is not guaranteed or endorsed by the publisher.

## Supplementary material

Supplementary Video 1 Paroxysmal events in Subject 1. Multiple events were recorded during two different hospitalizations. The semiology of the events was relatively stereotyped and consisted of dystonic facial grimacing, posturing of the upper arms, eye blinking, hand automatisms, and hyperkinetic behavior without a clear loss of contact. Scalp EEG recording did not reveal any associated ictal abnormalities during these events nor any change in the background despite over 50 events per day, which was inconsistent with epilepsy. Furthermore, interictal EEG was normal. These events were considered epileptic for a long time; however, in light of these recordings, a movement disorder related to the patient92s PLPHP deficiency is suspected.
