# Supplementary material for: Case report: PLPHP deficiency, a rare but important cause of B6-responsive disorders: A report of three novel individuals and review of 51 cases
Source: Front Neurol. 2022 Oct 17;13:913652. doi: 10.3389/fneur.2022.913652 (PMC9618642; doi:10.3389/fneur.2022.913652)
Supplement: Supplementary file 2 [file Table_1.docx]

Supplementary Material

# Appendix A. TRIZ Parameter Correspondence Table for CMEC Services

| TRIZ parameters | | | Service quality determinant | | Effects |
| --- | --- | --- | --- | --- | --- |
| 35 | Adaptability or versatility | **1** | | Service flexibility | Institutions should provide additional elderly care services as required by CMEC |
| 27 | Reliability | **2** | | Service reliability | Staff should provide reliable CMEC services to the elderly |
| 13 | Stability of object composition | **3** | | Service continuity | Reliable and regulated CMEC services for the elderly (e.g. routine health check-ups, monitoring of physical and physiological conditions, food delivery and bathing assistance) are required. |
| 24 | Loss of information | **4** | | Information symmetry | Clear communication from physicians and nursing staff is needed to ensure clients understand CMEC services |
| 35 | Adaptability or versatility | **5** | | Service adaptability | Institutions should provide CMEC services for the elderly according to their individual care needs |
| 29 | Manufacturing precision | **6** | | Service accuracy | The service delivery model should precisely meet the needs of CMEC with minimal waste |
| 17 | Temperature | **7** | | Healthcare workers’ attitude | Nursing staff should demonstrate friendliness, respect and understanding toward the elderly |
| 17 | Temperature | **8** | | Psychological and social support | Institutions should provide psychological and social support (e.g. offer mental health counseling and social activities). |
| 17 | Temperature | **9** | | Proactive service | Healthcare workers should be proactive in caring for the health status of the elderly |
| 35 | Adaptability or versatility | **10** | | Healthcare workers’ skills and knowledge | Knowledgeable, experienced, and skilled staff are needed to effectively handle the various problems faced by the elderly |
| 22 | Loss of energy | **11** | | Cost | The elderly should not have to spend more than expected when purchasing care services |
| 25 | Loss of time | **12** | | Waiting time | The elderly should not have to wait for long periods for the provision of services |
| 26 | Amount of substance | **13** | | Healthcare worker numbers | Institutions should provide adequate numbers of experienced healthcare workers |
| 33 | Ease of operation | **14** | | Convenience | Institutions should provide convenient CMEC services |
| 35 | Adaptability or versatility | **15** | | Selectivity | Institutions should provide a diverse range of CMEC services |
| 11 | Pressure or stress | **16** | | Efficiency and functionality of services | Institutions should use new equipment to improve service efficiency and functionality |
| 35 | Adaptability or versatility | **17** | | Customization | Institutions should provide customized CMEC services for the elderly based on their needs |
| 26 | Amount of substance | **18** | | Service capacity | There should be sufficient resource capacity for CMEC services |
| 24 | Loss of information | **19** | | Information transparency | The elderly and their families should have immediate access to information about care services (including the costs, purposes and procedures of services, and information about medications and side effects) |
| 9 | Speed | **20** | | Time of service delivery | Healthcare workers should control their work and rest time to ensure the prompt arrival of CMEC services at the homes of the elderly |
| 12 | Shape | **21** | | Environmental comfort | Institutions should provide a comfortable and satisfactory living environment for the elderly |
| 30 | Object affected harmful factors | **22** | | Safety and privacy | Institutions should provide a safe and private environment for the elderly |
| 14 | Strength | **23** | | Healthcare workers’ patience | Healthcare workers should be patient in answering the elderly’s questions and providing long-term care services (including massage, rehabilitation, and nursing services) |
| 38 | Extent of automation | **24** | | Service automation | Institutions should use automated equipment (including telemedicine equipment, intelligent body scanner, etc.) for CMEC services |
| 34 | Ease of repair | **25** | | Service compensation | Institutions should provide additional compensation services to the elderly who lose rights due to operational failures |
| 32 | Ease of manufacture | **26** | | Expertise | Healthcare workers must be proficient in all expertise provided by CMEC services |
| 39 | Productivity | **27** | | Efficiency | On-time service completion is required for group services and single services within a fixed period of time |
| 23 | Loss of substance | **28** | | Waste of resources | Institutions should assess CMEC services and provide appropriate services to meet the changing needs of the elderly without the waste of resources |
| 33 | Ease of operation | **29** | | Equipment convenience | Institutions should provide staff with equipment that is easy to handle and maintain |
| 36 | Device complexity | **30** | | Equipment complexity | Medical devices for CMEC should not have unnecessary and complex features |
| 28 | Measurement accuracy | **31** | | Equipment Accuracy | Measuring equipment must accurately analyze the health status of the elderly to ensure that physicians can make accurate diagnostic and treatment decisions |
| 13 | Stability of object composition | **32** | | Equipment stability | All medical equipment provided must be performance-ready at all times |
| 34 | Ease of repair | **33** | | Equipment maintenance | Medical equipment must be regularly maintained and updated |
| 33 | Ease of operation | **34** | | Ease of operation | Medical equipment interfaces must be ergonomically designed and user-friendly for elderly users |
| 8 | Volume of a stationary object | **35** | | Scope of services | Institutions must provide sufficient CMEC services to meet their scope of services |

*This table was compiled with reference to the TRIZ parameter correspondence table for aging-in-place services by Shie ^[12]^, which was combined with the needs of CMEC to form a TRIZ parameter correspondence table for CMEC services.

# Appendix B. RPN Assessment Scale for CMEC Service Failures

This questionnaire is designed to gather all the views of frontline staff and managers of elderly care institutions. Please select what you think is the best option from the questions below. Each question has three dimensions:

Severity (i.e., whether the impact of such failures is severe or not). **A severity rating of 1 indicates that the service failure had almost no impact, and 5 indicates the most severe service failure**.

Probability of occurrence (i.e., the likelihood that such failures will occur). **A** **probability rating of 1 indicates that it is almost impossible for the service failure to occur, and 5 indicates that the failure is bound to occur.**

Detectability (i.e., the level of your or your unit’s ability to detect and deal with a service failure). **A** **detectability rating of 1 indicates that you or your unit has the highest ability to detect a service failure, and 5 indicates that they cannot detect the service failure**.

The questions shown in this questionnaire do not mean that your unit necessarily has the service failure mentioned, and the questionnaire is anonymous, so please feel free to fill it in. Thank you for your cooperation.

F1: Failure of nursing staff to detect sudden illness in the elderly in a timely manner [matrix question]*

Causes: Inadequate healthcare workers, and prolonged interval between ward rounds
Effects: Failure leading to aggravation of the elderly person’s condition

|  | **1** | **2** | **3** | **4** | **5** |
| --- | --- | --- | --- | --- | --- |
| **Severity** | ○ | ○ | ○ | ○ | ○ |
| **Probability of occurrence** | ○ | ○ | ○ | ○ | ○ |
| **Detectability** | ○ | ○ | ○ | ○ | ○ |

F2: Failure of resident physician to reach the elderly person's bed in time (within three minutes) when the elderly person was taken ill [matrix question]*

Causes: Inadequate healthcare workers, prolonged arrival time of healthcare workers at beds
Effects: Inability of elderly care institutions to provide responsive services

|  | **1** | **2** | **3** | **4** | **5** |
| --- | --- | --- | --- | --- | --- |
| **Severity** | ○ | ○ | ○ | ○ | ○ |
| **Probability of occurrence** | ○ | ○ | ○ | ○ | ○ |
| **Detectability** | ○ | ○ | ○ | ○ | ○ |

F3: Failure of healthcare workers to judge a medical condition [matrix question]*

Causes: Inexperienced healthcare workers
Effects: Failure leading to missed optimal treatment window and death of the elderly

|  | **1** | **2** | **3** | **4** | **5** |
| --- | --- | --- | --- | --- | --- |
| **Severity** | ○ | ○ | ○ | ○ | ○ |
| **Probability of occurrence** | ○ | ○ | ○ | ○ | ○ |
| **Detectability** | ○ | ○ | ○ | ○ | ○ |

F4: Inadequacy of equipment provided by the institution for disease detection [matrix question]*

Causes: Inadequate maintenance of testing equipment, and inadequate service resources

Effects: Failure to meet the needs of the elderly, and failure causing illness in the elderly

|  | **1** | **2** | **3** | **4** | **5** |
| --- | --- | --- | --- | --- | --- |
| **Severity** | ○ | ○ | ○ | ○ | ○ |
| **Probability of occurrence** | ○ | ○ | ○ | ○ | ○ |
| **Detectability** | ○ | ○ | ○ | ○ | ○ |

F5: Failure of ambulances from higher level hospitals to arrive on time for elderly people with serious illnesses requiring referrals [matrix question]*

Causes: Inadequate healthcare workers, and prolonged ambulance arrival times

Effects: Failure leading to the aggravation of the elderly person’s condition and missing the optimal treatment window

|  | **1** | **2** | **3** | **4** | **5** |
| --- | --- | --- | --- | --- | --- |
| **Severity** | ○ | ○ | ○ | ○ | ○ |
| **Probability of occurrence** | ○ | ○ | ○ | ○ | ○ |
| **Detectability** | ○ | ○ | ○ | ○ | ○ |

F6: Inappropriate rehabilitation services/inability to tailor post-illness rehabilitation services to the individual [matrix question]*

Causes: Inadequate service resources (e.g., equipment, funding, and rehabilitation staff), and impatience of nursing staff
Effects: Failure leading to the degeneration and atrophy of physical functions in the elderly

|  | **1** | **2** | **3** | **4** | **5** |
| --- | --- | --- | --- | --- | --- |
| **Severity** | ○ | ○ | ○ | ○ | ○ |
| **Probability of occurrence** | ○ | ○ | ○ | ○ | ○ |
| **Detectability** | ○ | ○ | ○ | ○ | ○ |
